# Implementation of two high through-put techniques in a novel application: detecting point mutations in large EMS mutated plant populations

**DOI:** 10.1186/1746-4811-5-13

**Published:** 2009-10-07

**Authors:** Antoine LF Gady, Freddy WK Hermans, Marion HBJ Van de Wal, Eibertus N van Loo, Richard GF Visser, Christian WB Bachem

**Affiliations:** 1 Wageningen UR, Plant Breeding, Wageningen University and Research Center, PO box 386, 6700 AJ Wageningen The Netherlands; 2 Graduate School Experimental Plant Sciences, Building RADIX - West (building nr 107), Droevendaalsesteeg 1, 6708 PB Wageningen, The Netherlands; 3 Nunhems Netherlands BV, PO Box 4005, 6080 AA Haelen, The Netherlands

## Abstract

**Background:**

The establishment of mutant populations together with the strategies for targeted mutation detection has been applied successfully to a large number of organisms including many species in the plant kingdom. Considerable efforts have been invested into research on tomato as a model for berry-fruit plants. With the progress of the tomato sequencing project, reverse genetics becomes an obvious and achievable goal.

**Results:**

Here we describe the treatment of *Solanum lycopersicum *seeds with 1% EMS and the development of a new mutated tomato population. To increase targeted mutant detection throughput an automated seed DNA extraction has been combined with novel mutation detection platforms for TILLING in plants. We have adapted two techniques used in human genetic diagnostics: Conformation Sensitive Capillary Electrophoresis (CSCE) and High Resolution DNA Melting Analysis (HRM) to mutation screening in DNA pools. Classical TILLING involves critical and time consuming steps such as endonuclease digestion reactions and gel electrophoresis runs. Using CSCE or HRM, the only step required is a simple PCR before either capillary electrophoresis or DNA melting curve analysis. Here we describe the development of a mutant tomato population, the setting up of two polymorphism detection platforms for plants and the results of the first screens as mutation density in the populations and estimation of the false-positives rate when using HRM to screen DNA pools.

**Conclusion:**

These results demonstrate that CSCE and HRM are fast, affordable and sensitive techniques for mutation detection in DNA pools and therefore allow the rapid identification of new allelic variants in a mutant population. Results from the first screens indicate that the mutagen treatment has been effective with an average mutation detection rate per diploid genome of 1.36 mutation/kb/1000 lines.

## Background

Generating new genetic variation by mutagenesis in plants for the unraveling of biological processes and for the alteration of agronomic traits was viewed with great optimism in the mid sixties [1]. Later, this optimism was tempered by the complexity of using such mutants in classical breeding practice due to the difficulties in the identification of which mutation in a plant displaying an altered trait was responsible for the phenotype [2]. The development of plant molecular biology and biochemistry has facilitated the identification of individual genes insight into their function using reverse genetic tools such as antisense (PTGS) or RNAi [3-5]. These technologies opened up the possibilities of developing molecular tools for crop improvement. Although some GMO approaches have shown promising results, the regulatory framework and the consumer preferences makes the marketing of such products difficult. The advent of TILLING (Targeted Induced Local Lesions IN Genomes) has allowed the pinpointing of mutations in genes of interest [6]. TILLING combines mutagenesis protocols with PCR and a method for single nucleotide DNA polymorphism detection. In the original protocol, mutational detection combined an enzymatic digestion with a single strand specific DNA-nuclease and a high resolution denaturing polyacrylamide gel electrophoresis [6]. The first TILLING population was directed at the model plant *Arabidopsis thaliana*. However, there is no methodological restriction in the technique to model plants and large complex genomes proved equally amenable to the TILLING process [7-19].

Tomato (*Solanum lycopersicum L*.) is an important vegetable crop with numerous uses as fresh and processed produce and with a high nutritional value [20]. Tomato breeding has been successful in developing a wide variety of specialized cultivars for a number of markets (salad, cherry, beef and processing). Due in part to its commercial importance and its relatively uncomplicated genetics (diploid, self-compatible) there has been a large volume of molecular genetics produced on tomato connecting genes with traits of interest. Nevertheless, tomato breeding remains challenged by a series of problems that include disease/pest resistance and stress tolerance as well as numerous other targets directed at fruit quality and plant architecture [21]. For these breeding aims, targeted mutations in genes connected to traits of interest creating knockouts or modified activities may provide useful material.

EMS mutated populations have already been created for different tomato cultivars. One was developed on the M82 line in Israel [22]. The Micro-Tom [23] miniature tomato cultivar was also EMS mutagenised, first in the French National Institute of Agricultural Research (INRA) and later in the University of Tsukuba [24].

Despite numerous improvements in the protocol and the use of different mismatch-specific and sensitive endonucleases like ENDO1 [25], TILLING is still a labor intensive and therefore costly process. In this paper we present two high throughput technologies that have been adapted to tilling in plants together with the characterization of a large tomato EMS mutated population.

## Results and discussion

### EMS induced mutant population development

**Figure 1:**
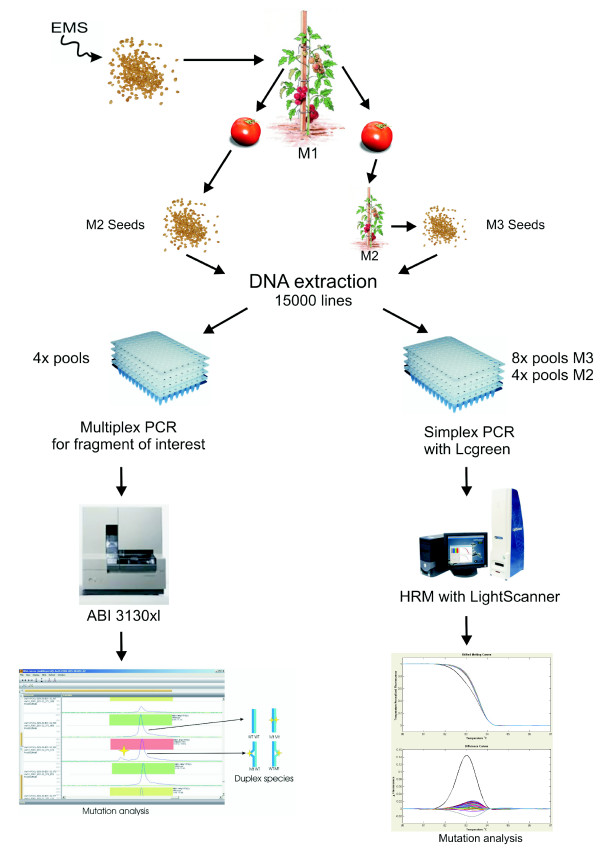
**Mutant production and identification using the TILLING process**. M2 population, 10 seeds originating from the first M1 fruit were ground and ultimately DNA was isolated, the M2 population comprises 8225 lines. M3 population, from the second M1 fruit, 8810 lines were grown and selfed, seeds were harvested for 7030 lines and a seedlot subset (10 seeds) was used for DNA extraction. For both M2 and M3 population, DNA was pooled 4 or 8 fold, depending on the selected screening method: CSCE; After Multiplex PCR amplification with fluorescent labelled primers, samples are directly pooled together and loaded on capillaries filled with CAP polymer. Pools containing a mutation are identified using Applied Maths' HDA peak analyser software. HRM; Following PCR amplification in presence of LC-Green+™, pools are analysed for their product melting temperatures.

EMS is a chemical mutagen predominantly inducing C to T and G to A transitions randomly throughout the genome [26]. In 2006, *Solanum lycopersicum *seeds cv. TPAADASU were treated with EMS. M1 plants were grown in the field in Italy. The M1 population was the starting point for the creation of two mutant populations. For each M1 mutant plant two fruits were harvested and their seeds kept separate. The first population, named M2 population, is composed of 8810 families. Of these, 585 families were eliminated due to low seed set. However, any seeds that that were obtained have been stored for future propagation and DNA extraction. The second population, grown from the seeds of the second M1 fruit was grown as M2 families and 5 fruits for each of the 8810 families were harvested. A set of 7030 seed lots were harvested from the M2 fruits and stored in a seed bank. This second population was named M3 population. Development of the M2 and M3 populations is schematically represented in figure 1 together with the screening platforms. For both populations, DNA was extracted from a pool of 10 seeds originating from each seed lot. For mutation screening purposes, this DNA was 4× or 8× flat pooled.

**Figure 2:**
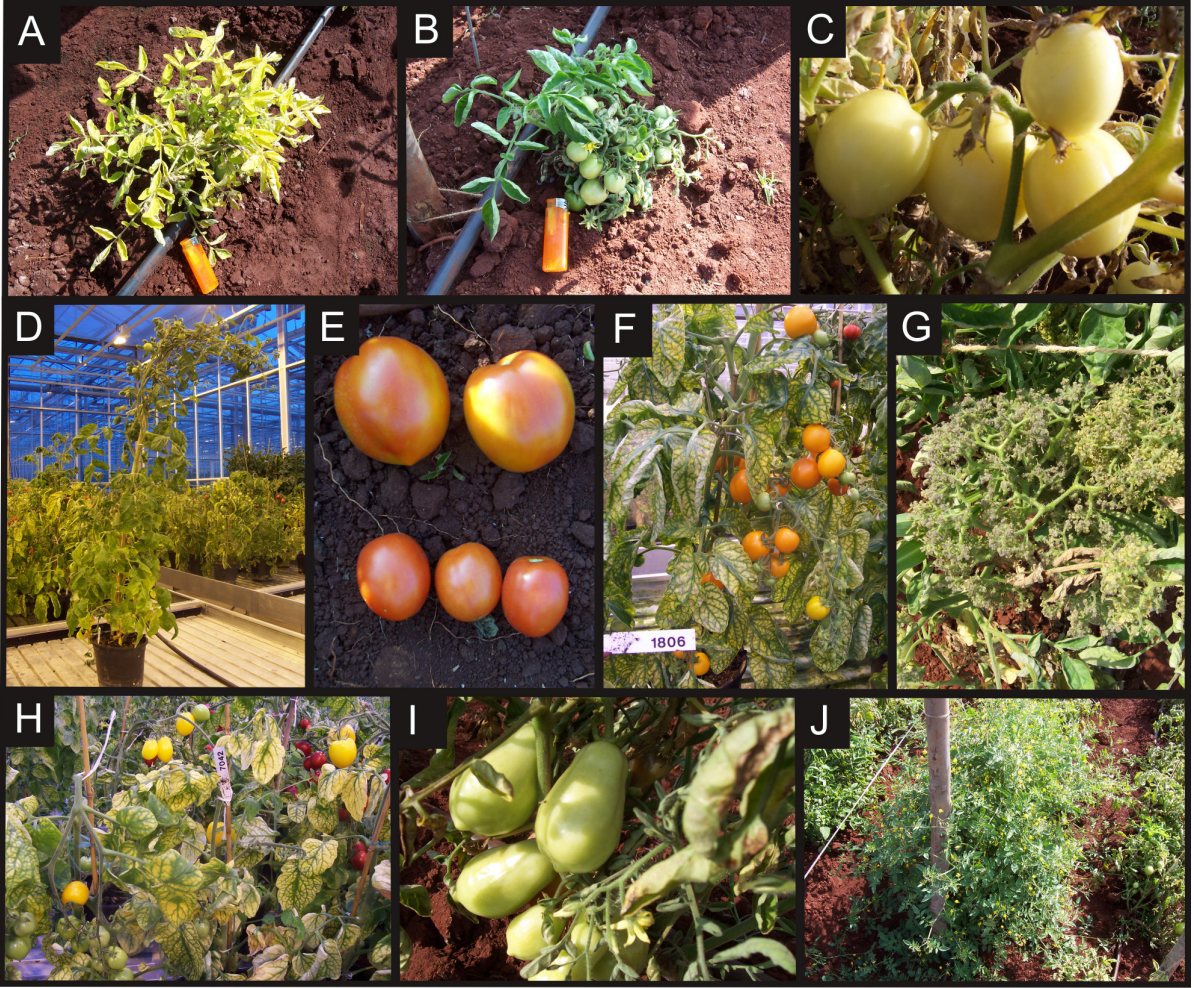
**Examples of tomato mutant traits in the M2 population**. Lines affected for plant architecture, (A) dwarf chlorophyll deficient plant, (B) small bushy plant, (D) oversized plant with indeterminate growth and absence of fruit grapes. Lines affected for flower and fruit color and size, (C) fruit color: Light colored ripened fruits, (E) fruit size: small fruits compare to *wt *on top of the picture, (F) fruit color: orange fruits, (G) inflorescence structure: *Anantha*-like mutant, (H) fruit color: bright yellow fruits, (I) fruit shape: egg-shaped fruit and (J) *S. pimpenellifolium *plant.

In the field, the population showed a broad range of mutant phenotypes for fruit color, shape and size. Plant architectural traits, such as plant size, branching, leaf shapes, inflorescences and flower organization were also highly variable in the M2 plants (Figure 2).

### Adaptation of high-throughput SNP detection platforms to mutation screening

The standard system for point mutation discovery in TILLING projects is based on an endonuclease enzyme, either CEL1 or ENDO1, which specifically cleaves at the mutation point by recognizing mismatches in double stranded DNA molecules. Pools containing a mutation within the fragment of interest are visualized on denaturing polyacrylamide gels using a Li-Cor DNA analyser [6].

Adapting efficient SNP discovery methods developed for the human genetics [27,28],, we have implemented two novel mutation detection screening techniques termed Conformation Sensitive Capillary Electrophoresis (CSCE) [29] and High Resolution Melt curve analysis (HRM) [30] which have proven to be sensitive and high-throughput methods in human and plant genetics [31]. An overview of the screening process is schematically represented in Figure 1.

#### Conformation Sensitive Capillary Electrophoresis

**Figure 3:**
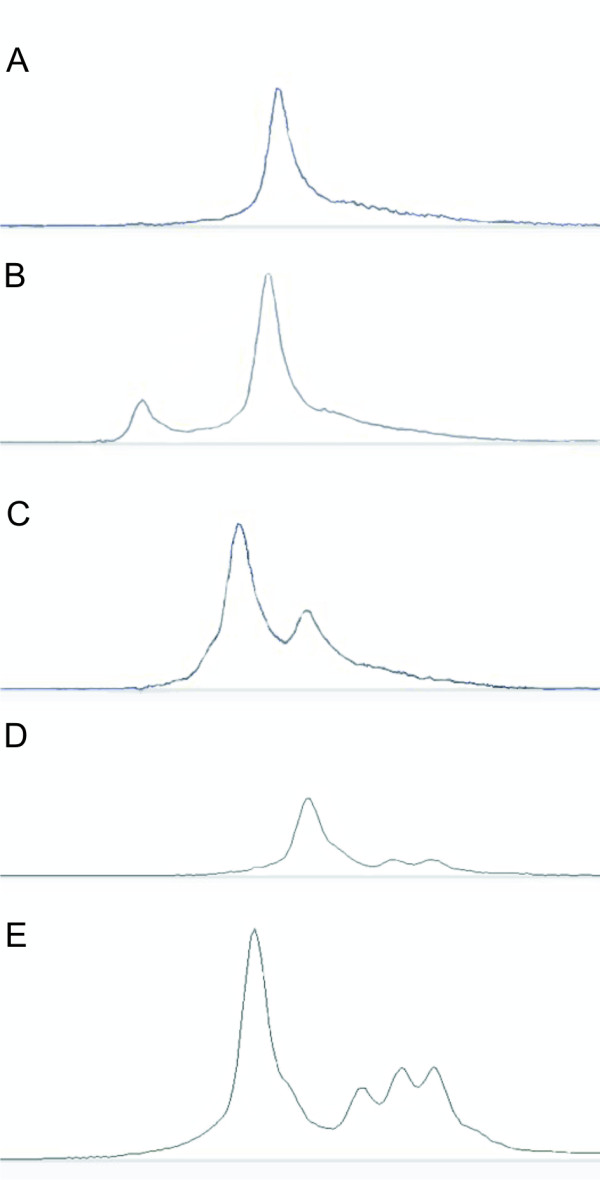
**Output peaks from CSCE screen**. (A) Negative control peak, represents a pool not containing any mutation, all the fragments migrate through the capillary at the same speed. (B) Positive control peak represents a pool containing DNA isolated from *S. pimpenellifolium *seeds. The fragments forming heteroduplexes have a different motility through the CAP polymer than the majority of the other products. The homoduplexes in this example run faster. C, D & E; Examples of peak patterns different from either the positive or the negative controls and therefore identified as mutant, one line in each of these pools contain a mutation. Direct sequencing confirmed the results obtained with CSCE. (A) C2_At4g11570 negative control; (B) C2_At4g11570 Positive control; (C) C2_At4g11570 mutant; (D) *Expansin1 *mutant (E) Second *Expansin1 *mutant

CSCE is a non-enzymatic differential DNA conformation technique for SNP discovery. When considering a DNA pool containing a mutant allele as well as wild-type alleles, following PCR, the amplified product will anneal randomly to other fragments that may or may not contain a mutation. Thus several duplex species will be formed: (i) homoduplexes resulting from the annealing of wild-type/wild-type or mutant/mutant fragments together and (ii) heteroduplexes resulting from mutant/wild-type fragments annealing together. Because of mismatches that are formed, the heteroduplexes will migrate at a different speed than the homoduplexes during electrophoresis in ABI 3130 × L capillaries filled with CAP, a semi-denaturating polymer, thus allowing the identification of pools containing a mutation within the targeted fragment. Presence of heteroduplexes is identified as an altered peak shape as illustrated in Figure 3.

To set up the screening platform, 7 DNA fragments were selected from the SGN marker database based on identified SNP's between *S. lycopersicum *and *S. pimpenellifolium*. DNA from the two species was mixed in different ratios ranging from 1 in 2 to 1 in 32. After PCR on the dilution series, PCR fragments were loaded on the capillaries and results showed that when *S. pimpenellifolium *DNA was 16 times diluted, the SNP was still detectable as differentially migrating fragments in CSCE (data not shown). Although these results demonstrate that 8 fold pooling was feasible in diploid material, a pooling of 4 times was used in order to avoid missing mutations.

Using CEL1/ENDO1 - Li-Cor platforms, it is possible to screen for fragments up to 1.5 kb in one run [32]. In CSCE, however, target fragment lengths between 200 to 500 bp are optimal [27]. In our experience, this limitation can be compensated for by multiplex PCR of up to 4 fragments and by using forward primers labelled with 3 different dyes for each product labelling. Combining multiplex PCR and multiple labelling with amplicons differing in size, it is possible to screen for 12 fragments in one run by pooling all PCR products post-PCR reaction. For example, three genes are targeted; four fragments per gene can be screened. Four multiplex PCRs can be performed in each of these, one fragment from each gene is amplified. Finally, all products from the four multiplex PCRs are pooled together and run altogether in CSCE.

Since neither of the two technologies explored here (also see HRM below) allow the screening of large target amplicons, we therefore elected to focus only on coding regions of the genome. Furthermore, we initially analysed coding regions with the CODDLE software [33] to identify target fragments rich in residues that are more likely to give rise to STOP codons [34]. Other factors that were considered is, the location of regions coding for conserved protein domains, as these coding regions are the most likely to affect the activity of the protein (e.g. binding domains in transcription factors or active centres in enzymes). To localise such putative sites, the amino acid sequence of candidate genes can be analysed with the NCBI Conserved Domain Database (CDD) and using tailor made software such as SIFT [35].

#### High Resolution Melt curve analysis (HRM)

**Figure 4:**
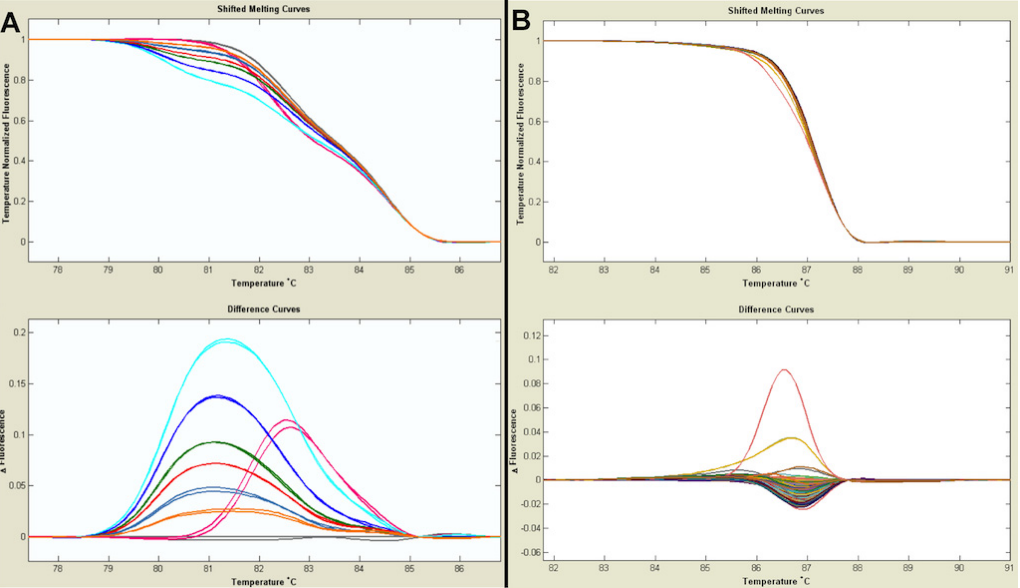
**Output data from HRM analysis (A and B)**. The upper panel shows the fluorescence change in dependence on the temperature. The lower panel shows the relative difference in melting curves compared to a reference sample. Decrease in fluorescence reflects the annealing state of the duplex species in the sample. Samples starting to melt at a lower temperature are likely to contain a SNP within the amplified fragment. (A) These graphs are presented the data from the serial dilution experiment. A PCR product (TG581 RFLP marker (SGN-M84)) has been amplified from *S. lycopersicum *(grey), *S. pimpenellifolium *(pink) or dilution of *S. pimpenellifolium *in *S. lycopersicum *with the following ratios: 1/1 (light blue); 1/3 (dark blue); 1/7 (green); 1/9 (red); 1/15 (blue); 1/31 (orange). (B) HRM screening output from screen performed on 4× pools from the M2 population for the *PSY *gene (PSY-1 fragment; table 1). The pink melting curve corresponds to a pool containing a C to T mutation within the amplified fragment, position and type of mutation was identified by Sanger sequencing.

As with CSCE, HRM is a non-enzymatic screening technique [28]. During the PCR amplification, LCgreen Plus+™ molecules are intercalated between each annealed base pair of the double stranded DNA molecule. When captured in the molecule, the LCgreen Plus+™ emits fluorescence at 510 nm after excitation at 470 nm. A camera in the LightScanner^® ^apparatus records the fluorescence intensity while the plate is progressively heated. At a temperature dependant on the sequence specific stability of the DNA helices, the double stranded PCR product starts to melt, releasing the LCgreen Plus+™ dye. The release of dye results in decreased fluorescence that is recorded as a melting curve by the LightScanner^®^. Pools containing a mutation form heteroduplexes in the post-PCR fragment mix. These are identified as differential melting temperature curves in comparison to homoduplexes (Figure 4).

We repeated the same strategy used when setting up the CSCE platform in order to test whether HRM was suitable for large scale mutation screening. Fragments containing SNP's between *S. lycopersicum *and *S. pimpenellifolium *were amplified on a dilution range of *S. pimpenellifolium *in *S. lycopersicum *from 1 in 2 to 1 in 32. It was possible to distinguish heteroduplex formation even when *S. pimpenellifolium *DNA was 32 times diluted (figure 4). An 8 fold flat pooling of the population was therefore undertaken in order to start with the analysis of mutations in candidate genes.

### Mutation screening in candidate genes and false positive/negative estimation

In *Arabidopsis thaliana*, increased proline is correlated with an increased tolerance to abiotic stresses. Antisense suppression of the expression of a proline degradation enzyme, proline dehydrogenase was shown to lead to elevated proline levels in various tissues and increased tolerance to freezing and salt stress [36]. To create similar phenotypes in tomato, without the use of genetic modification, the *ProDH *gene [DFCI *Solanum lycopersicum *Gene Index: TC209088 TC589] was chosen for mutational screening using HRM. Screening of 6,700 M3 families for the proline dehydrogenase (*ProDH*) gene on a total length of 788 bp, led to the identification of 8 mutations.

**Figure 5:**
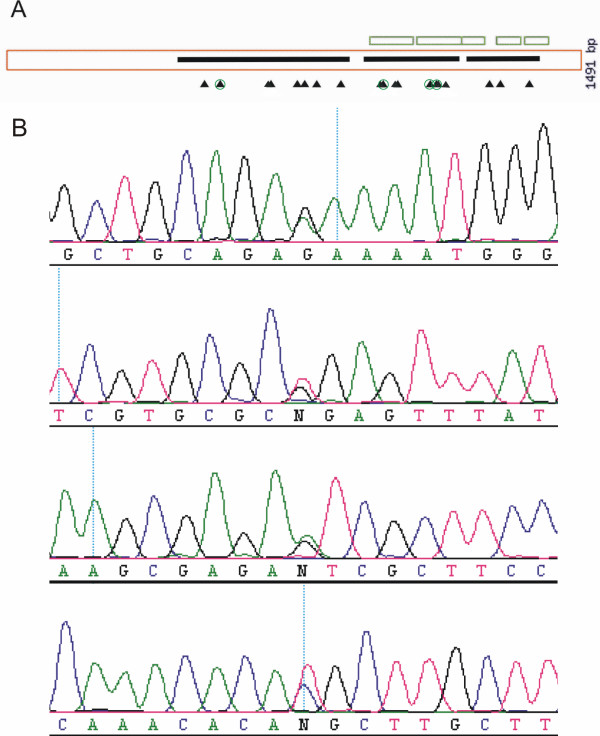
**PARSESNP output for the *ProDH *gene following mutation identification in both M2 and M3 mutagenised populations**. (A) PARSESNP graphical positioning of identified SNPs. The orange box represents the *ProDH *coding sequence. The black triangles represent the position of the mutations. Black stripes represent the PCR fragments that were analysed with HRM. (B) Following high-throughput mutation screening, *ProDH *putative mutant families were sequenced. G to A and C to T transitions are identified as double peaks. In total 19 mutations were identified. Here four chromatograms displaying mutations are shown as examples, they correspond to the green circled arrow heads in (A).

The *ProDH *gene was also screened using HRM on the 8025 families of the M2 population. Using 8-fold (8×) pooled DNA, only four mutations were identified. It was expected that the mutation frequency in the M3 and M2 populations would be equal; however, surprisingly the M2 had a more than two fold lower mutation frequency. To eliminate the possibility that this difference was due to a too high dilution in the M2 screen, the screening was repeated on 4 fold (4×) pools. With 4× pools 12 mutations were identified. All mutation events were mapped on the *ProDH *cDNA sequence (Figure 5). The analysis of results from the two M2 screens using 8× and 4× pooled DNA with the same set of genotypes, showed that of the 4 mutations found with 8× pooling, 3 were also detected with 4× pooling. We conclude that one mutation was not detected using 4× pooling. This yields a rough estimate for the false negative rate in 4× pooling of 0.25 (s.e. = 0.217). This value is probably an overestimation due to the low number of observations (Table 1). In contrast, the false negative rate of 8× pooling with the M2 samples is significantly higher than with 4× pooling based on a binomial test, (P < 0.001). Of the 12 detected mutants with 4× pooling, 9 were not detected with 8× pooling, yielding a false negative rate of 0.75 with an s.e. = 0.125 (Table 1). From these results we estimate the true mutation frequency is 2.5 SNPs per Mb. The mutation detection rate is however lower and depends on the pooling strategy. No such false negative rate is available for the M3 screening.

**Table 1:** *ProDH *screen results

	**M2 4x (a)**	**M2 8x (b)**	**M3 8x (c)**
Total # lines screened (N)	8025	8025	6692

# of positive pools after Screen 1 (on pools) (P1)	87	33	23

# of positive individuals after Screen 2 (on individuals from positive pools) (P2)	47	32	27

# of confirmed mutations after sequencing (P3)	12	4	8

# detected as % of # of followed up after Screen 1 (D1)	13.80%	12.10%	34.80%

			

# of false negatives (no mutant detected while present) (F1)	1	9	No data

False negative rate (fraction) (F1-f)	0.25	0.75	No data

Mutation detection frequency (mutations per 1000 kb amplicon length) MD1	1.9	0.63	1.5

True mutation frequency (mutations per 1000 kb amplicon length) M1	2.53	2.53	No data

Explanations of variable names: in the description the general variable name is given (e.g. P1). To indicate the value in the table, indices a, b and c have been used to indicated the column. Calculation details: P1, P2, P3, F1 were direct results. D1 = P3/P1 (expressed as %), e.g. D1a = P3a/P1a = 12/87 = 13.8%. F1a-f = F1a/P1b (expressed as fraction = 1/4 = 0.25; F1b-f = F1b/P1a (expressed as fraction = 9/12 = 0.75. MD1 = P3/(N*amplicon length) = (12/(8025 * 788)) * 1,000,000 = 1.9 per 1,000 kb; M1a = MD1a/(1-F1a-f) = 1.9/(1-0.25) = 1.9/(0.75) = 4 * 1.9/3 = 2.53; M1b - MD1b/(1-F1b-f) = 0.64/(1-0.75) = 0.63/(0.25) = 4 * 0.63/1 = 2.53

**Figure 6:**
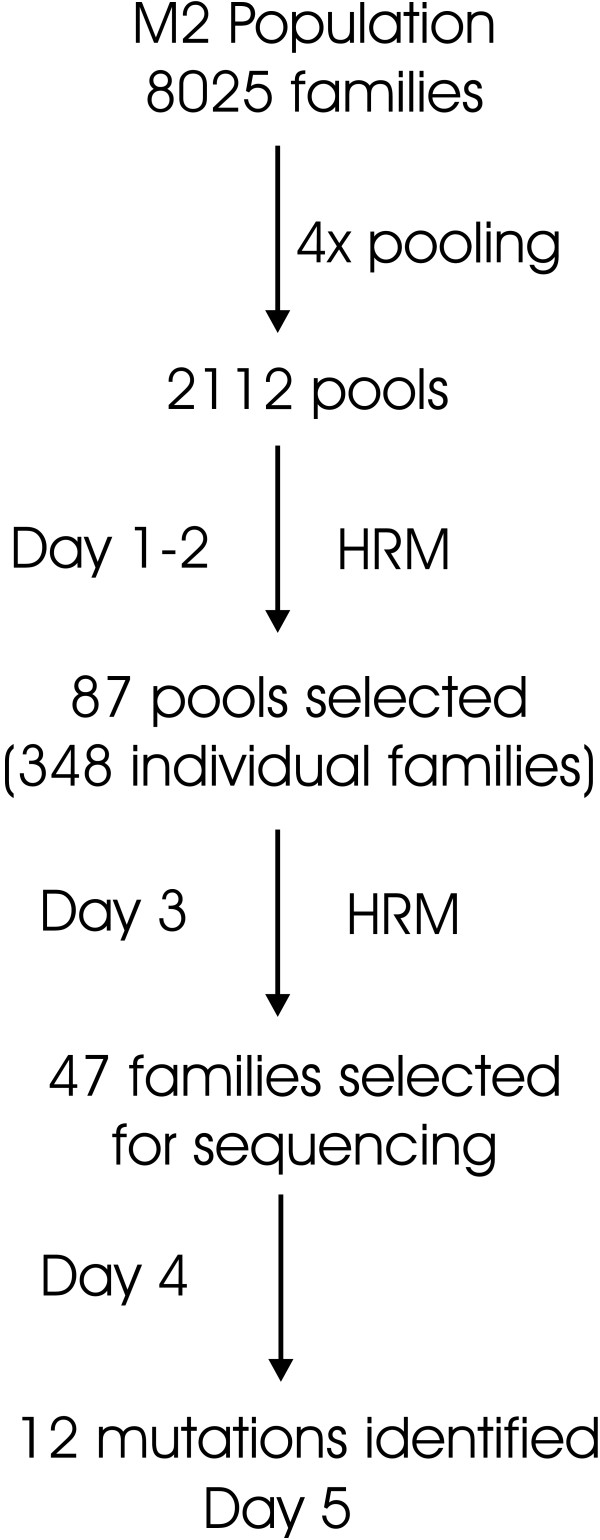
**Overview of the mutation screen for *ProDH *on the M2 population**. 8025 EMS mutated families from the M2 population were four-fold flat pooled providing 2112 pools divided among 22 plates of 96 wells. Following PCR, pools were analysed for HRM with a LightScanner^® ^apparatus. HRM analysis identified 87 pools as putatively containing a mutation. Following deconvolution and PCR, the HRM analysis was repeated and 47 single mutant families were sent for Sanger sequencing. Finally based on sequence data 12 mutations were identified. From the first PCR till the identification of the mutants with sequence data, the work load was a total of 5 working days for one person as shown on the left side of the arrows.

The selection criteria used for identifying potential mutation events in the screening of the pools is based on a visual evaluation of the HRM curves. These pools are deconvoluted to individuals in a second HRM screen and only a subset of these are selected for sequencing which provides the final validation of the HRM results (Figure 6). For the HRM technology there is a clear linkage to the work load and the rate of false negative and false positives. In order to minimize false negatives, a large number of false positives are accepted in the first screen (pooled samples). Although there is a significant reduction of samples in the screen on the individual (families), the final appraisal of the mutations is made on the basis of the sequence of the amplicons.

### Mutation frequency of the mutant populations

The seven selected SNP-fragments used to set up the CSCE screening platform were screened on 2300 individual families. In this screen, 5 mutations were identified and confirmed by direct sequencing (data not shown). This initial screen validated the CSCE technique and confirmed that the technique was promising for mutation detection. After this pilot experiment, the first gene screened for mutations was the Auxin Response Factor 7 (*ARF7*). For this, the coding region was divided in 3 fragments. CSCE and subsequent sequencing data confirmed that 5 mutations within these amplicons could be identified using the 5000 M3 families.

Screening for mutants in additional genes (Phytoene synthase (PSY) a gene involved in the synthesis of the compounds responsible for the characteristic tomato red color; Sucrose synthase 2 (Sus2) an enzyme involved in sugar metabolism during the early development of tomato fruits) using both detection technologies resulted in the identification of a total of 44 mutations over 35.2 Mb coding DNA, indicating an effective mutagen treatment for our population with an average mutation density of 1 mutation every 737 Kb (Table 2). Using CSCE and HRM as mutation screening techniques, the fragment size that can be analysed is limited to a maximum of 500 bp [27,37], therefore we exclude introns from the screens as mutations in these regions are generally neutral.

**Table 2:** Tomato TILLING results overview.

**Product**	**Size (Kb)**	**Screened families**	**Mutations identified**	**Mutation detection rate (n mutations/1000 kb)**	**Mutation density (1/n kb)**
**SNP Markers**	1.467	2300	5	1.48	675

***ARF7***	0.894	5000	5	1.12	894

***ProDH M3***	0.788	6700	8	1.52	660

***ProDH M2***	0.788	8025	12	1.90	527

***PSY***	0.804	8025	8	1.24	807

***Sus2***	0.812	8025	6	0.92	1086

**All products**	0.8514	38075	44	1.36	737
	**(weighted average)**	**(independent amplicons)**			

Mutation frequencies on the tomato genome were calculated as follow: ((total size of amplicons) × (total number of screened lines))/(number of identified mutations). The average mutation frequency was calculated as: ((weighted average amplicon size) × (total number of independent amplicons))/(total number of mutations).

Mutation screens in the other tomato populations show similar frequencies as the one observed in the present study: Saito and co-workers [24] estimated their mutation frequency to be of 1 mutation event per Mb screened. Moreover in a recent study using direct sequencing with 454 GS FLX Rigola and co-workers [38] identified 2 mutations in 889 bp of the *Sle*IF4E gene in 3008 M2 families of the M82 population, resulting in a mutation density of 0.75 mutation per 1000 Kb screened. In our population, we observe a slightly higher mutation frequency: 1.36 mutation per 1000 Kb screened. Therefore we suggest that a useful mutation density has been achieved in our tomato mutant population for breeding purposes. In addition, considering that creating a valuable mutant population is a compromise between high mutation rates and minimising the risks of sterility and early development defects, choice of 1% EMS treatment of the seeds and a 60% survival rate was acceptable. We subsequently observed a loss of 10% of the mutant families in the two successive generations, because of defects in germination and plantlet development, flower development or parthenocarpy. We therefore conclude that the mutagen treatment was optimal for our purposes.

For practical breeding the mutation detection rate is the most relevant information as it determines how many families/genotypes have to be screened before a mutant will be found. However, when designing mutation breeding also the real mutation rate is important, as this determines the amount of non-target genes that will be affected. Taking the false negative rate with *ProDH *of the M2-set into account gives a true mutation frequency of 2.53 mutations per 1000 kb or 1 mutated nucleotide in 351 kb as compared to the mutation detection rate of 1.9 mutations per 1000 kb or 1 nucleotide in 527 kb (Calculations are based on the screen of the *ProDH *gene on the M2 population 4 fold pooled; see tables 1 and 2). It is clear that an accurate measurement of the false negative rate is important. This cannot be judged only on the basis of the detection limit of the method, here HRM. Our results show a detection limit for a mutation <1:31 (see dilution with SNP), however with 8× pools we still miss some mutants at a dilution of 1:16. In literature [39-42], generally only mutation detection rates rather than true mutation rates are reported as usually the false negative rate is not determined.

Our complete set of mutant plant material comprises ~ 15,000 families. These families may be regarded as two independent M2 and M3 populations (Figure 2). We argue that these two populations are different in that they originate from two different M1 fruits. The treatment of seeds with EMS results in chimerism in the M1 generation. During development, the apical meristem will form all the aerial part of the plant. Each cell or group of cells from this meristem is from the very early stage of development predestined to form specific organs, thus cell fate determination predates the effect of EMS treatment. Within the embryonic apical meristem a number of cells will give rise to the production of the gametes. Canales *et al*. [43] state that plant germ-lines arise late in the development from archesporial initials in the L2 layer of the anther and ovule primordia, indicating that various apical meristems already present before the EMS-treatment in the seed may contribute to gametes, thus giving rise to various independent mutations in the M1-plant. Irish and Sussex [44] cite various authors stating that in an apical meristem the 'genetically effective cell number' (GECN, independent cells contributing to gamete production) is only 2 to 3 cells in *Arabidobsis*, which means that in each inflorescence only a limited number of independent mutations will be present. In barley, a minimum of 6 shoot sectors (coming from different apical meristems) are already initiated in the seed, as determined from leaf mutant analysis and these shoot sectors each will give an independent floral spike for which already one or two cells are present per spike at the moment of the EMS treatment that will give rise to gametes [45]. Therefore, in barley, at least 6-12 independent sporogenous cells are present in the seed before the mutation treatment. No such information was found for tomato, but it may be concluded that also in tomato, a single plant will have multiple initial cells in the seed before the EMS-treatment is applied and therefore will produce multiple independent mutations in its offspring. Also, it is probable that offspring arising from a single fruit will have a high probability of carrying the same mutation as apparently generally in plants only 2-3 initial cells form the gametes in an inflorescence.

It is therefore likely that two fruits coming from two different parts of a plant will contain different sets of mutations. That this is indeed the case was shown by screening the *ProDH *gene on the M2 as well as on the M3 population. With the HRM platform we identified 11 new mutations in the M2 population that are different from the 6 found in the M3 population and in both collections only two common mutations were identified. These results confirm that our two populations (see Figure 2) are independent mutant populations.

To find common mutations within the M3 and the M2 population is not unexpected. Indeed, as two fruits were randomly harvested from the M1 plants, it is probable that in some cases they were collected from the same inflorescences. In a single inflorescence, it is more probable to retrieve two cells both originating from an identical gamete stem cell mutated by the EMS treatment. Thus, two different fruits may have partial similarities for mutation content. Another, perhaps even more plausible explanation is the existence of background mutations in the M0-plants. With background mutations it is easy to explain both results 1) that the same mutant sequences were found in M2 and M3-families in 2 out of 8 mutations and 2) the fact that homozygous mutants were found (3 out of 19 different mutations were homozygous for the M2 and M3-family analysis with *ProDH*).

### General conclusions on the screening platforms

We show that both CSCE and HRM SNP detection methods are very interesting and cost effective developments with regard to high-throughput mutation screening methods or experiments. The standard endonuclease-Li-Cor platform, despite being more sensitive in terms of heteroduplex detection in pooled samples [40] than the techniques described here, is also labor intensive due to the numerous steps involved in the procedure: PCR, enzymatic reaction, gel preparation and loading and visual analysis. Here we have developed two platforms only requiring preparations of PCR prior to sample analysis. Using the LiCor/ENDO1 platforms it is possible to screen 3000 plants per day for an average target length of 1 kb [42]. However, using CSCE 2.5 kb of target sequences could be screened on 1200 families per day which for the ease of comparison correspond to 3000 families screened for 1 kb target sequence in a day. CSCE throughput can be increased by the use of 48 or 96 capillaries instead of 16 capillaries ABI analyzer used here. With HRM one 96-well plate can be processed in less than five minutes and results analyzed in another five minutes. Depending on the availability/capacity of PCR machines in our laboratory, we screened 4600 families for 1 kb of target sequence per day. This throughput allowed us to screen 1 kb of coding sequence in less than 5 days on 8025 families starting from screening the 4× DNA pools till confirmation of the mutation by Sanger sequencing (Figure 6). Here also the throughput could be increased by using 384 well plates. Both the CSCE and HRM platforms are easy to set-up, cost effective and high-throughput technologies that in our opinion out-perform the other mutation scanning methods published so far.

## Materials and methods

### Plant material and EMS treatment

TPAADASU, a highly homozygous inbred parental line, used in commercial processing tomato breeding, was selected for mutagenesis treatment with the following protocol. After seed germination on damp Whatman^® ^paper;  for 24 h, ~20,000 seeds, divided in 8 batches of 2500 respectively, were soaked in 100 ml of ultra pure water and ethyl methanesulfonate (EMS) at a concentration of 1% in conical flasks. The flasks were gently shaken for 16 h at room temperature. Finally, EMS was rinsed out under flowing water.

Following EMS treatment, seeds were directly sown in the greenhouse. Out of the 60% of the seeds that germinated, 10600 plantlets were transplanted in the field.

From the 8810 M1 lines that gave fruits, two fruits per plant were harvested. DNA was isolated from seeds coming from the first fruit, constituting the M2 population DNA stock.

From the second fruits, 10 seeds were sown in the greenhouse to produce M2 plants, of which 3 plants were grown in open field. During harvest, five fruits from one M2 plant per line were collected. M3 seeds were isolated from the M2 fruits and 10 of the seeds were used for DNA isolation and constitute the M3 population DNA bank (Figure 1).

*S. pimpenellifolium *(Accession number LA121) plants were grown in the field along with the M2 lines (Figure 2). One *S. pimpenellifolium *was planted every 96 mutant lines. DNA was obtained and stored in 96 well plates in the order the lines were planted in the field. Thus, one well from each DNA stock plate contains *S. pimpenellifolium *DNA and this position varies for each stock plate, A1 for plate number 1, B1 for plate number 2 etc. As many SNPs can be found between *S. lycopersicum *and *S. pimpenellifolium*, this setup was designed to provide controls for the plate identification (specific position for each stock plate as described above) and positive controls for the screening platforms.

TPAADASU is a commercial breeding line and may be obtained for non-commercial research purposes from the originator: Nunhems BV.

### High throughput seed DNA extraction

Per mutant line, 10 seeds were pooled in a Micronic^® ^deepwell tube;  from a 96 deep-well plate, 2 stainless balls were added to each tube. The tubes and seeds were frozen in liquid nitrogen for 1 minute and seeds were immediately ground to a fine powder in a Deepwell shaker (Vaskon 96 grinder, Belgium; ) for 2 minutes at 16,8 Hz (80% of the maximum speed). 300 μl Agowa^® ^Lysis buffer P from the AGOWA^® ^Plant DNA Isolation Kit  was added to the sample plate and the powder was suspended in solution by shaking 1 minute at 16,8 Hz in the Deepwell shaker. Plates were centrifuged for 10 minutes at 4000 rpm. 75 μl of the supernatant was pipetted out to a 96 Kingfisher plate using a Janus MDT^® ^(Perkin Elmer, USA; ) platform (96 head). The following steps were performed using a Perkin Elmer Janus^® ^liquid handler robot and a 96 Kingfisher^® ^(Thermo labsystems, Finland; ). The supernatant containing the DNA was diluted with binding buffer (150 μl) and magnetic beads (20 μl). Once DNA was bound to the beads, two successive washing steps were carried out (Wash buffer 1: Agowa wash buffer 1 1/3, ethanol 1/3, isopropanol 1/3; Wash buffer 2: 70% ethanol, 30% Agowa wash buffer 2) and finally eluted in elution buffer (100 μl MQ, 0,025 μl Tween).

Grinding ten *S. lycopersicum *seeds produced enough DNA to saturate the magnetic beads, thus highly homogenous and comparable DNA concentrations of all samples were obtained. Comparing with lambda DNA references, a concentration of 30 ng/μl for each sample was estimated. Two times diluted DNA was 4 fold flat pooled. 2 μl pooled DNA was used in multiplex PCRs for mutation detection analysis.

### Fragments of interest

PCR fragments known to contain SNP's between *S. lycopersicum *and *S. pimpenellifolium *were used to set up the CSCE mutation detection platform. Six COSII markers from the Tomato-EXPEN 2000 v52 map were chosen from the SOL Genomics Network (SGN) website because containing polymorphism between *S. lycopersicum *and *S. pimpenellifolium*: C2_At4g11570 (chromosome 8); C2_At5g63840 (chromosome 4); C2_At3g11710 (chromosome 6); C2_At1g03150 (chromosome 6); C2_At1g09340 (chromosome 6)) and C2_At4g24690 (chromosome 6). A RFLP marker from the same map was also selected from the SGN database: TG581 (SGN-M84). New primers were designed to target a shorter fragment than the RFLP marker, their sequences are as follow: forward primer CGGTAATCCGTTCAACGTCC; reverse primer TTGGTCTTTAAAACATGGCGC. In addition to this set of markers, part of the *Expansin1 *gene was used for the set-up of the polymorphism screening methods and for the mutation frequency estimation. These seven fragments were divided in 3 multiplex groups. The first group contains: C2_At4g11570 and C2_At5g63840. The second group is composed of: C2_At1g03150 and C2_At4g24690. In the last group: C2_At1g09340, C2_At3g11710 and the *Expansin1 *fragment. For each of the fragments, the forward primers were ordered with 5' fluorescent labels: 6FAM™ for C2_At4g11570 and C2_At1g03150; VIC^® ^for C2_At5g63840 and C2_At4g24690; NED™ for: _At1g09340, C2_At3g11710 and *Expansin1 *(Table 3). Primers for the SGN COSII markers can be found on the SGN website .

**Table 3:** Target genes and associated primer sequences.

**Gene**	**Product size**	**Primer sequences**	
		
**Region**		**Forward primers (5' - 3')**	**Reverse Primers (5' - 3')**
***Auxin Response Factor 7***			
ARF7_1	307 bp	AATTCAGAGTTATGGCACGCTTG	TCGAGAACTCCCAGCCAATATGTG
ARF7_2	418 bp	CCTCCTCCCATAAGTTGTATGAAAC	ACAGTGTTACCCCATTAGTAGTTCC
ARF7_3	469 bp	TCCTTGCTGCTGCTGCTCATGC	GCAGGAAGGGCTGTACTATGACCAC

***Expansin 1***			
Exp_1	307 bp	TACAGCCAAGGATACGGAGTT	ATGGGATCCTGCGATAAGTT

***Phytoene Synthase***			
PSY-1	220 bp	CATGGAATCAGTCCGGGAGGGA	CTTCACCAAGGCTGCCTGCC
PSY-2	366 bp	TCCTCCCTTTTTCTCCACTTCAAGC	AAGCCCTCAGCAAAAGTGACATCA
PSY-3	368 bp	TTCAATAGCGTAATTGTCTAACCTTCCA	ACCGGATAACCGAAGAGCTCA

***Proline Dehydrogenase 2***			
ProDH_1	403 bp	GTGGAACATGCCACCGATAATGAATC	TCAATTGCAGGTTGAATGGTTGTG
ProDH_2	301 bp	AAAGATGATCAGCCTTTGATATTCGG	CCTGATTCAATGTTATGAGTGGCGAG
ProDH_3	234 bp	AAAACTTGCTGCAACCAAAGCTATAG	TGTCGAATGCCGATGTAGACAGCATG

***Sucrose Synthase 2***			
Sus2-1	463 bp	TTTTCTGTGGAGTGAGGATTGTAGCTAA	GATAAGAGGAAAAACACCACAGACCTGC
Sus2-2	449 bp	TCGTGTGAAGAATTTAACCGGACTTGT	TTGCATTTCAAAGAAATCAGCTAGCA

*ARF7: Auxin Response factor 7*, GenBank: EF121545.1; *Exp_1: Expansin 1*, GenBank: U82123.1; *PSY: Phytoene Synthase *GenBank: EF157834.1; *ProDH: Proline Dehydrogenase*, TC209088: ; *Sus2; Sucrose Synthase 2*, GenBank: AJ011535.1

Multiplex PCR conditions were as follows for all the CSCE screens: 94°C, 3 minutes; 35 cycles for the following steps: 94°C, 5 seconds; 55°C, 30 seconds; 72°C, 90 seconds and a final 10 minutes at 72°C. PCR conditions for HRM screens are described in the High Resolution Melt curve analysis part.

Following set up of the platforms, four target genes were screened using one of the developed techniques. PCRs were conducted in one or several regions of each gene using the primers described in Table 3.

### Screening platforms

#### A. Conformation Sensitive Capillary Electrophoresis (CSCE)

Multiplex PCR reactions were performed in 10 μl volume with 0.15 ng, 4 times pooled genomic DNA. Labeled primers were added to the PCR mastermix to a concentration 5 times lower (1 μM) than that of the unlabeled primers. Post PCR, samples were diluted 10 times. Before the CSCE run, 2 μl of the diluted products were added to 38 μl of MQ water.

The samples were loaded on 50 cm capillaries (injection time and voltage: 16 seconds, 10 KVolts; Run voltage: 15 KVolts) from the ABI 3130 × l (Applied Biosystems, USA, ) apparatus filled with semi-denaturating polymers of the following composition: 5 g Conformation Analysis Polymer (CAP) (Applied Biosystems, 434037, 9%), 2,16 g Ureum, 0,45 g 20xTTE (national diagnostics, EC-871), completed with MQ water up to 9 g. The running buffer was prepared with 1× diluted TTE and 10% glycerol. The oven temperature was set to 18°C[27].

Raw data were analysed with the HeteroDuplex Analysis (HDA) software from BioNumerics (Applied-maths, Belgium, ), The program differentiates peak patterns of hetero-duplexes (mutant) and homo-duplex molecules (wild type) thus providing the possibility of selecting DNA-pools containing an individual line mutated in the target gene.

#### B. High Resolution Melt curve analysis (HRM)

The LCgreen PCRs were performed on 8× flat pools in FramStar 96-wells plates (4titude, UK, ) with the following conditions: 94°C, 2 minutes; 40 cycles, 94°C, 5 seconds; fragment dependent Tm, 10 seconds; 72°C, 10 seconds; a denaturation step of 30 seconds at 94°C and renaturation by cooling to 30°C. 2 μl (15 ng) of pooled DNA was mixed with 2 μl of F-524 Phire™ 5× reaction buffer (FINNZYMES, Finland, ), 0.1 μl Phire™ Hot Start DNA Polymerase (FINNZYMES, Finland), 1 μl LCGreen™ Plus+ (BioChem, USA), 0.25 μl of 5 mM primers, and completed to 10 μl with MQ water) according to manufacturer recommendations. Pools containing a mutation were screened using a LightScanner^® ^System (Idaho Technology Inc., USA, ). Positive pools were selected by analyzing the melting temperature profiles; when the pool contains a mutation it will show a lower melting temperature.

## Competing interests

The authors declare that they have no competing interests.

## Authors' contributions

ALFG was responsible for the development of both screening platforms; the DNA isolation; the screening of the candidate genes; the data analysis and drafted the manuscript. FH actively participated in the development of the CSCE platform. ENVL was responsible for the statistical analysis. MHBJVW was responsible for the population development; oversaw the pipeline development. CWBB and RGFV oversaw the pipeline development and helped draft the manuscript. All authors have read and approved the manuscript.
